# Predicting hERG repolarization power at 37°C from recordings at room temperature

**DOI:** 10.1002/ctm2.1266

**Published:** 2023-05-18

**Authors:** Barbara B. R. Oliveira‐Mendes, Malak Alameh, Jérôme Montnach, Béatrice Ollivier, Solène Gibaud, Sylvain Feliciangeli, Florian Lesage, Flavien Charpentier, Gildas Loussouarn, Michel De Waard, Isabelle Baró

**Affiliations:** ^1^ Nantes Université CNRS INSERM l'institut du thorax Nantes France; ^2^ Labex ICST Université Côte d'Azur INSERM Centre National de la Recherche Scientifique Institut de Pharmacologie Moléculaire et Cellulaire Valbonne France

Dear Editor,

Loss‐of‐function and gain‐of‐function mutations in the *KCNH2* gene cause long and short‐QT syndromes (LQTS or SQTS), respectively, predisposing to life‐threatening cardiac arrhythmias.^1^, ^2^ *KCNH2* encodes the voltage‐gated K^+^ channel hERG that generates the delayed rectifier K^+^ current I_Kr_ controlling the action potential (AP) duration.^3^ Prolonged or shortened ventricular AP durations are visualized as abnormal QT interval duration on the electrocardiogram. The occurrence and severity of *KCNH2*‐related arrhythmias are determined by the variant functional impact. Sequencing *KCNH2* has provided a plethora of variants associated or not with pathological cardiac phenotypes and indexed in the ClinVar NCBI database. Discriminating pathogenic variants from benign ones would clarify the genetic background of patients and relatives, and stratify the risk of adverse events. In the face of a wide spectrum of hERG functional defects, we looked for a way to summarize the net loss or gain of function in a unique index. We defined the repolarization power as the time integral of the K^+^ current (I_hERG_) developed during an AP clamp.^4^

To be meaningful, the repolarization power, representing *in‐vivo* hERG contribution to repolarization, has to be established at physiological temperature. However, the patch‐clamp success rate, defined as maintaining a seal resistance above a given threshold, is low at physiological temperature which significantly limits the high‐throughput characterization of channel variants by automated patch‐clamp systems (Supporting Information results).^5^

Here, to succeed in fast‐track functional characterization of hERG variants using a high‐throughput patch clamp, we modified the standard AP‐clamp protocol to establish, at room temperature, a repolarization power index, predicting the reference one measured at 37°C. We also show that the repolarization power determined at room temperature is predictive for 2 pathogenic hERG variants with different biophysical dysfunctions.

Decreasing the recording temperature affects hERG biophysical processes,^6^, ^7^, ^8^ and thus should affect the repolarization power. In HEK293 cells stably expressing wild‐type (WT) hERG, both I_hERG_ evoked by a ventricular AP (Supporting Information, Figures S1 and S2; 1A) and the deduced repolarization power (Figure 1C) indeed decreased with temperature. In order to extrapolate the reference (i.e. at 37°C) repolarization power of hERG channels from recordings at room temperature, we need to compensate for the temperature effects on I_hERG_ kinetics. So, we arbitrarily ‘dilated’ the time with a given factor on the AP protocol duration (Figure 1B, inset). With a time factor of 2, the current at 27°C had time to further develop (Figure 1B), and consequently, the repolarization power closely matched the one obtained with a factor of 1 at 37°C (Figure 1C and Figure S4).

**FIGURE 1:**
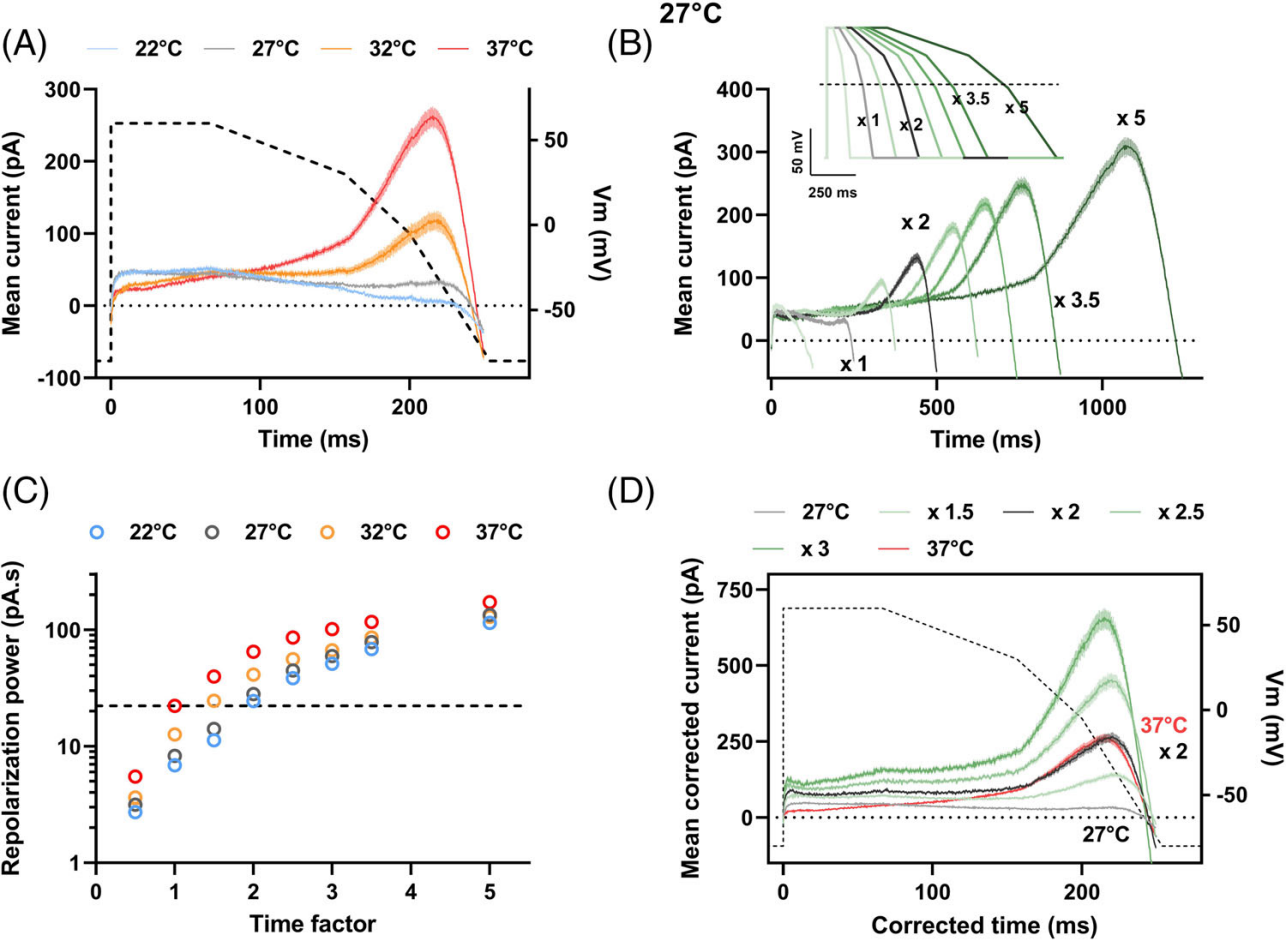
Repolarization power of wild‐type (WT) hERG as a function of temperature. A simplified and optimized action potential was applied (AP‐clamp) by an automated patch clamp system in 384‐well plates on HEK293 cells stably expressing hERG. (A) Mean (± SEM) current recordings during AP‐clamp at various temperatures (in pA, *n* = 194, 211, 114 and 172 cells at 22–37°C, respectively). Dashed line: AP time course (voltage scale: right Y axis). (B) Mean (± SEM) current recordings during AP‐clamp at 27°C for AP of various durations (see inset for APs; for currents: *n* = 168−254 for Time x 0.5 to x 5). The small inward current observed in (A) and (B), when the AP is returning to resting values, is attributed to contamination of the intracellular solution by the extracellular Tyrode solution, intrinsic to the cell catch process in the automated patch clamp (see supplementary information). (C) Mean (± SEM) time integral of the recorded currents: repolarization power, at various temperatures versus time factor (n = 155−232, 168−254, 79−133 and 144−173 at 22–37°C, respectively). Horizontal dashed line: repolarization power at 37°C: 22.3 pA.s. (D) As in (B), at 27°C, after time and current corrections using various factors from 1.5 to 3 on the respective recordings. For example, for the recordings obtained during the AP of 2 x duration, the time was divided by 2 and the current multiplied by 2. Note the overlap of the current corrected by the factor of 2 at 27°C (black) with the reference current obtained during the standard AP at 37°C (red).

When Vandenberg *et al.* investigated hERG temperature sensitivity, they calculated temperature coefficients (Q_10_, factors expressing the change observed when the temperature is increased by 10°C) of 1.4 for the whole‐cell conductance and 1.7 to 2.6 for activation, deactivation, inactivation and recovery from inactivation rates.^7^ We corrected kinetics and current amplitude using a single value. We observed, in two independent assays, that the factor of 2 was the most appropriate to fit with the 37°C data (Figure 1D and Figure S4). Hence, slowing the AP protocol by 2‐fold generates WT hERG current and repolarization power predicting, at 27°C, the ones obtained at 37°C. Switching to the conventional patch‐clamp technique on HEK293 cells transiently overexpressing WT hERG, we confirmed that I_hERG_ increased indistinctively by increasing temperature from 22 to 32°C or by increasing AP duration by a factor of 2 (Figure 2A,B). Again, the current density time courses were similar when recorded either at 32°C with a standard AP or at 22°C with a 2‐fold slowed AP (Figure 2C,D). The extracted repolarization power values were not significantly different (Figure 2E).

**FIGURE 2:**
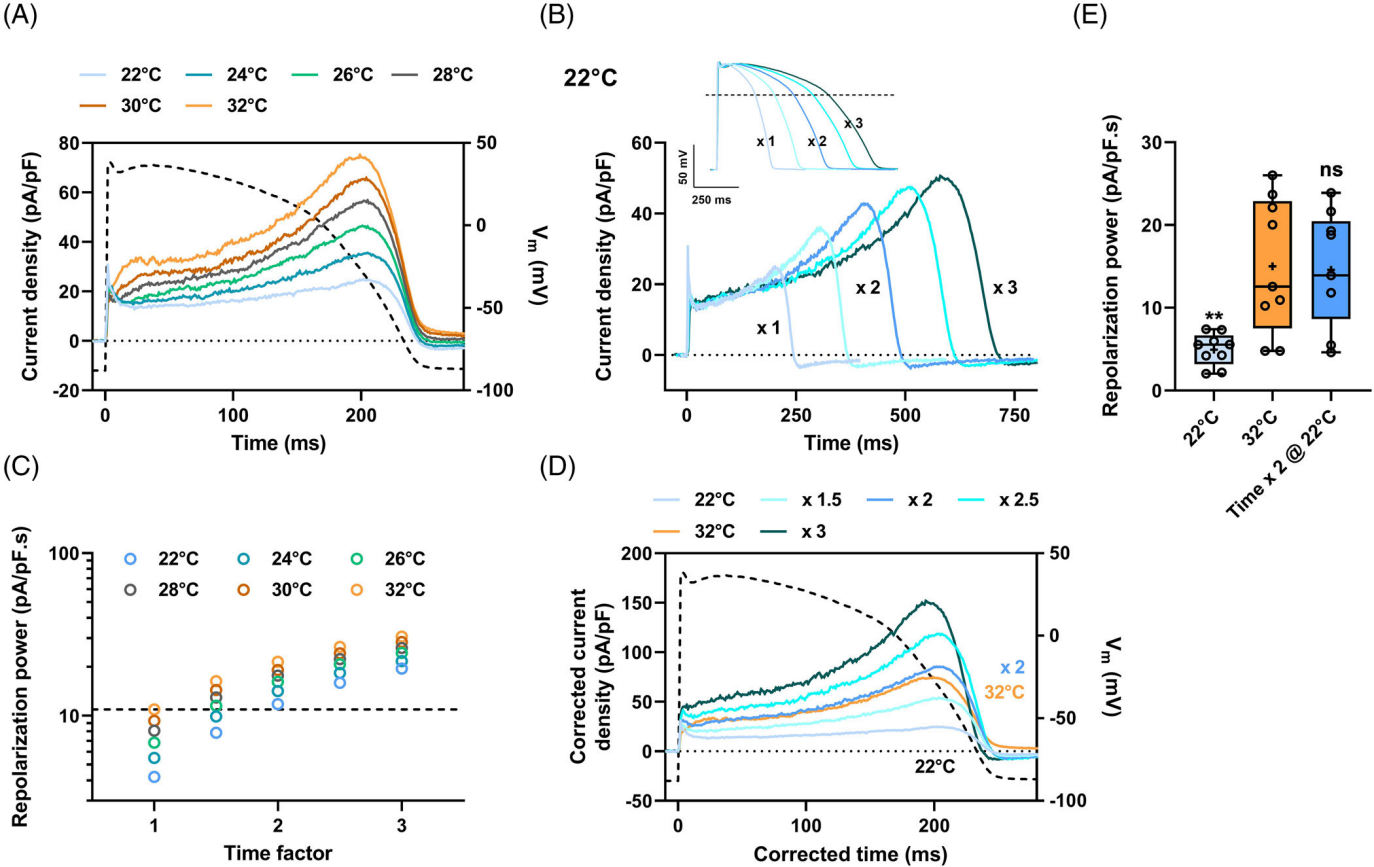
Repolarization power of wild‐type (WT) hERG as a function of temperature in HEK293 cells transiently expressing hERG. A model action potential was applied (action potential [AP]‐clamp) using a conventional patch clamp. (A) Typical current recordings on a single cell during AP‐clamp at various temperatures. Dashed line: time course of the model AP^31^ (voltage scale: right Y axis). (B) Current recordings during AP‐clamp at 22°C (same cell as A) for AP of various durations (inset). (C) Corresponding repolarization power at various temperatures *versus* time factor. Horizontal dashed line: reference repolarization power at 32°C: 10.9 pA/pF.s for this cell. (D) As in B, at 22°C, after time and current density corrections using various factors from 1.5 to 3 on the respective recordings. For the recordings obtained during the AP of x 2 duration, the time has been divided by 2 and the current density multiplied by 2. Again, note, as in Figure 1D, the overlap of the current at 22°C, corrected by 2 (blue), with the reference current obtained during the standard AP at 32°C (orange). (E) Tukey plot of the repolarization powers recorded at 32 and 22°C during the standard AP or AP of doubled duration (*n* = 9) *versus* 32°C: ns: non‐significant; **: *p* < 0.01.

Next, we estimated this correcting value in the case of 2 hERG mutations linked either to acquired LQTS (p.R328C) or to SQTS (p.D591H).^4^, ^9^ The same factor of 2 was obtained for the p.R328C hERG mutant (Figure 3A–C). Of note, its effect, when compared to WT, could clearly be detected on reference repolarization power predicted at 22°C (Figure S5B). As for WT and p.R328C, the p.D591H repolarization power values extracted from data at 32°C, and at 22°C using a factor of 2, were not significantly different (Figure 3F). However, I_hERG_ profiles were not fully overlapping: at 22°C, the corrected I_hERG_ time course was shifted toward the end of the AP (Figure 3E). But despite this shift, the mutant effect is still visible at 22°C on normalized corrected p.D591H current profile when compared to WT (Figure S5A–C).

**FIGURE 3:**
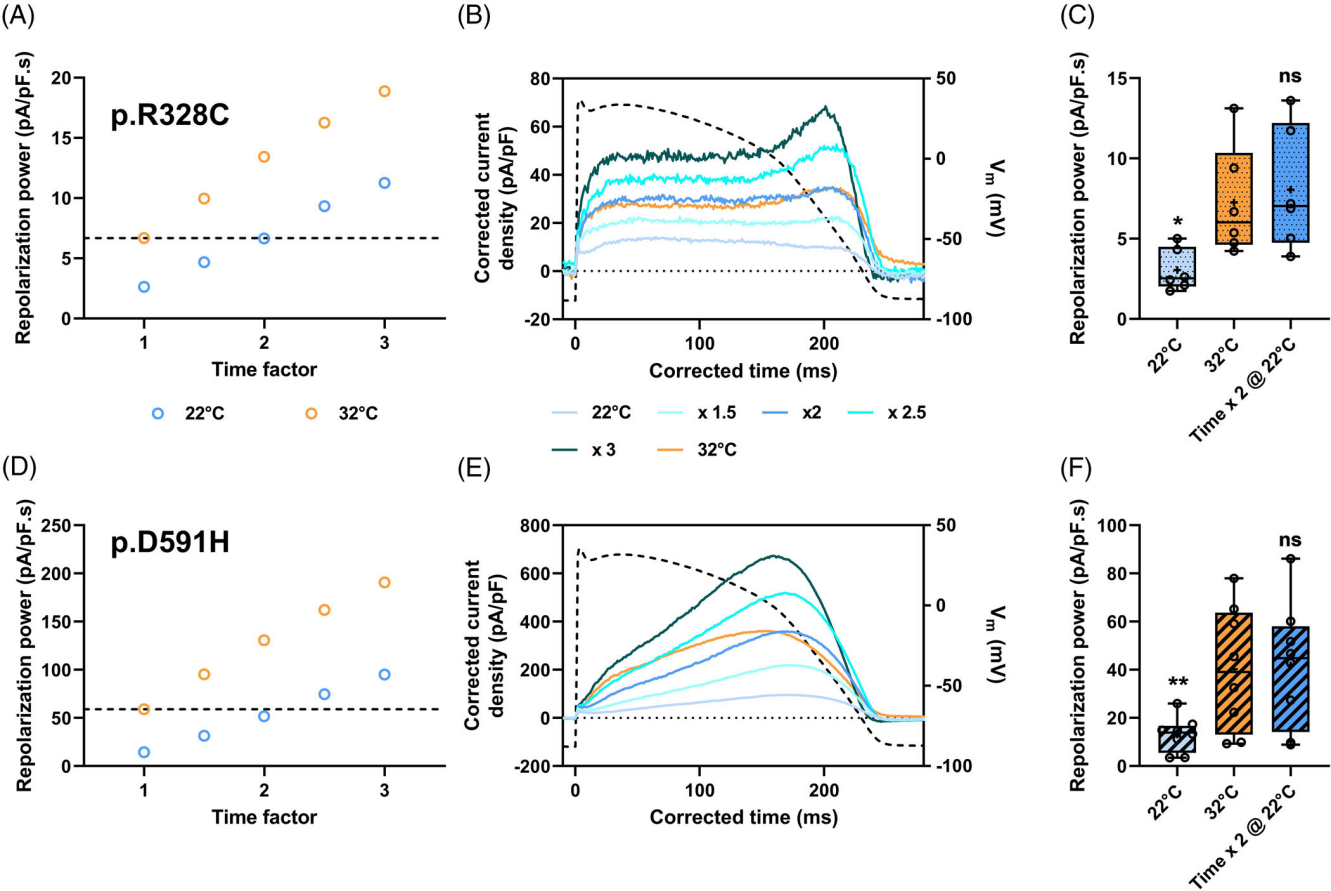
Repolarization power of long QT syndrome (LQTS) (A–C) and short QT syndrome (SQTS) (D–F) hERG as a function of temperature. Action potential clamp was applied (action potential [AP]‐clamp) on HEK293 cells transiently expressing p.R328C (A–C) or p.D591H (D–F) hERG variant. (A and D) Repolarization power at various temperatures *versus* time factor in typical cells. Horizontal dashed line: reference repolarization powers at 32°C: 6.7 and 59.1 pA/pF.s for p.R328C and p.D591H hERG, respectively. (B & E) Current recordings during AP‐clamp at 22°C (blue; same cells as A & D, respectively) for AP of various durations after time and current density corrections using various factors from 1.5 to 3 on the respective recordings; reference current recording during AP‐clamp at 32°C (orange). (C & F) Tukey plot of the repolarization powers recorded at 32 and 22°C during the standard or AP of doubled duration, for each variant (*n* = 6 and 8 cells for p.R328C and p.D591H, respectively) *versus* 32°C: ns: non‐significant; *: *p* < 0.05; **: *p* < 0.01.

The repolarization power is a time integral of a current time course. A specific and precise time frame during which I_Kr_ is contributing to cardiac AP repolarization is essential for proper AP termination, but not detected by this calculation. Thus, it is possible to get the same repolarization power as WT, despite changes in hERG channel kinetics. Therefore, it may be of interest to add a second indicator which will be the time at which I_hERG_ is maximal to automatically detect this behavior.

Altogether, these data indicate that a time factor of 2 may be used to efficiently determine the repolarization power at room temperature, highly similar to that developed at 37°C during the AP. We are aware that using a single correcting value to summarize the effects of temperature on hERG current is counterintuitive in regard to known temperature effects on various biophysical parameters.^6^, ^7^, ^8^ However, it appears to be reliable in the strictly limited context of the AP course.

Failure in the drug development process is mostly due to adverse effects on hERG current. Automated patch‐clamp systems using cell lines have upscaled the safety testing flow of novel drug candidates. However, it has been shown frequently that half‐maximal inhibitory concentration (IC_50_) may vary with the voltage protocol for drugs showing a voltage‐dependent inhibition.^10^ Repolarization power change at 37°C may give more relevant IC_50_ values, although replacing physiological temperature by time dilatation during AP‐clamp may not be straightforward, since binding and unbinding kinetics may not have the same temperature sensitivity as the repolarization power.

In summary, we present a simple, reliable and practical protocol to measure the reference repolarization power of a given hERG variant at room temperature, compatible with high throughput automated patch‐clamp system.

## CONFLICT OF INTEREST STATEMENT

The authors declare no conflict of interest.

## FUNDING INFORMATION

Fédération Française de Cardiologie: Grands projets – 2019 to MDW and FL; Agence Nationale de la Recherche, Grant/Award Numbers: ANR‐11‐LABX‐0015 and ANR‐21‐CE17‐0010‐CarDiag to MDW and FL; Fondation Leducq: ‘Equipement de recherche et plateformes technologiques’ to MDW; Conseil Régional des Pays de la Loire, Grant/Award Number: 2016−11092/11093 to MDW; European Regional Development Fund, Grant/Award Number: 2017/FEDER/PL0014592 to MDW; Fondation Lefoulon Delalande, Research Grant 2021 to BBRO‐M

## Supporting information

Supporting Information
