# Supplementary material for: Predicting hERG repolarization power at 37°C from recordings at room temperature
Source: Clin Transl Med. 2023 May 18;13(5):e1266. doi: 10.1002/ctm2.1266 (PMC10194418; doi:10.1002/ctm2.1266)
Supplement: Supplementary file 1 — Supporting Information [file CTM2-13-e1266-s001.pdf]

Predicting hERG repolarization power at 37°C from recordings at room temperature

Barbara B.R. Oliveira-Mendes[1], Malak Alameh[1,2], Jérôme Montnach[1], Béatrice Ollivier[1], Solène Gibaud[2], Sylvain Feliciangeli[2], Florian Lesage[2], Flavien Charpentier[1], Gildas Loussouarn[1,*], Michel De Waard[1,2,*], Isabelle Baró[1,*]

[1]Nantes Université, CNRS, INSERM, l'institut du thorax, F-44000 Nantes, France.
[2]Labex ICST, Université Côte d'Azur, INSERM, Centre National de la Recherche Scientifique, Institut de Pharmacologie Moléculaire et Cellulaire, Valbonne, France.

* These senior authors codirected this work.

Short running title: Fast determination of hERG repolarization power

Address for correspondence:
Isabelle BARÓ, PhD
l'institut du thorax, Inserm UMR 1087, CNRS UMR 6291
IRS-UN, 8 quai Moncousu
44007 Nantes cedex 1, France
e-mail: isabelle.baro@inserm.fr

Supplemental information

The duration of the QT interval on the surface electrocardiogram (ECG) is a representation of the repolarization time in cardiac ventricles. QT intervals vary as a function of various physiological (age, heart rate, hormones...) or pathophysiological (heart disease, fever, or drug intake) factors[1]. Normal QT intervals, when corrected by heart rate (QTc), are below 470-480 ms. When these QTc intervals are markedly prolonged, generally to values greater than 550–600 ms, polymorphic ventricular tachycardia such as torsade de pointes, elicited by emotions or physical activity, may occur and can degenerate to fatal arrhythmias such as ventricular fibrillation. Excessive QTc prolongations may have various origins: mutations in genes related to ion channels, as in the congenital long QT syndrome (cLQTS)[2], or upon exposure to stressing environmental conditions as in the acquired long QT syndrome (aLQTS)[3]. aLQTS is frequently provoked by the intake of QT-prolonging drugs, many of them of common use[4]. In this case, reversion back to normal generally follows withdrawal of the causative trigger.

Three genes encoding ion channel pore-forming subunits have been identified as responsible for up to 75% of the cLQTS cases: *KCNQ1* (K_V7.1 channel, 30–35% of LQTS cases), *KCNH2* (hERG *alias* K_V11.1 channel, 25–30%) and *SCN5A* (Na_V1.5 channel, 5–10%)[5–7]. A third of aLQTS patients carries cLQTS mutations, those on *KCNH2* being more common[8]. Physiologically, the two voltage-gated K^+ channels K_V7.1 and hERG, are responsible, as pore-forming channel α-subunits, for delayed outward currents, I_{Ks} and I_{Kr}, respectively, involved in the cardiac action potential (AP) repolarization[9]. Their regulation contributes to AP duration adaptation to heart rate. A reduction in outward current and/or an increase in inward current, a condition called reduced repolarization reserve[10], leads to AP duration lengthening, underlying the QT interval prolongation. About a thousand of cLQTS-associated *KCNH2* missense variants have been reported[11].

Finally, gain-of-function hERG mutants have been detected in patients with abnormally short QT duration (≤ 360 ms) on the ECG, presenting symptoms varying from atrial to ventricular fibrillation and sudden death[12,13].

For the last decades, sequencing *KCNH2* has provided a plethora of variants associated or not with clear pathological cardiac phenotypes[14,15]. Distinguishing pathogenic or likely pathogenic variants from the benign ones is a critical information to clarify the genetic background of LQTS patients as well as their relatives. Today, up to 85% of *KCNH2* detected missense variants are still classified as variants of unknown significance (VUS) or with conflicting interpretations[16] according to the American College of Medical Genetics and the Association for Molecular Pathology classification guidelines[17]. Various databases gathering data from LQTS patients have been continuously developed, as Bamacoeur in France[18], but they all have limited information regarding the functional impact of the variants at the molecular

level. In addition, when functionally investigated, the heterogeneity of the models and approaches prevents from clearly classifying them as illustrated by different studies focusing on the same variants [19–24]. In addition, the maximal hERG current (I_{hERG}) is often used as single index to quantify the loss or gain of function impact. Such an index may not capture the variation of some biophysical parameters such as a shift of the activation curve or of the inactivation curve because both gates are maximally open when the peak current is measured. Therefore, in an attempt to standardize the assessment of hERG variants molecular pathogenicity, we have recently designed a hERG phenotyping pipeline[24]. In order to speed up the molecular phenotyping, we designed a new voltage-clamp protocol to determine more than 10 biophysical parameters of hERG current in 35 seconds. To easily summarize the net functional effect of the modified biophysical parameters, we defined a new, simple and unique index, we called repolarization power, to grade the molecular pathogenicity of hERG channels instead of using only the maximal hERG current. This index is the time integral of the current or current density recorded during an AP-shaped voltage-clamp stimulation (AP-clamp), therefore proportional to the total amount of K^+ ions crossing the membrane during each AP.

The development of the automated patch-clamp allows now to studying up to 384 cells in parallel, boosting the variant functional investigation speed. However, the aforementioned repolarization power, representative of *in vivo* hERG contribution to repolarization, has to be established at physiological temperature. Indeed, the K^+ current conducted by hERG channels (I_{hERG}) depends on temperature but in a complex manner [25–28]. Recently, Lei and collaborators have developed a short and informative protocol to extensively study hERG behavior and its temperature dependence. Namely, they associated a simple Hodgkin-Huxley kinetic model, an Eyring formulation of the temperature dependence in the model, and a 15-s stimulation protocol (staircase protocol) designed for any patch-clamp set-up, including high-throughput automated systems[29,30]. All these studies showed that the relative occupancy of the channel open state (total K^+ conductance) and the rates of activation, deactivation, inactivation, and recovery from inactivation have different temperature sensitivities, the activation being far more temperature sensitive than inactivation[25,26,29,30]. Furthermore, Lei *et al.* also showed that experimental estimations of temperature coefficients (Q_{10}) are highly protocol dependent. Therefore, estimation of the effects of hERG variants on repolarization *in vivo* requires one to ideally work at 35-37°C.

On the other hand, as reported by Rajan and colleagues, in CHO, CV1, or HEK cells studied with the Nanion NPC-16 Patchliner Quattro, the patch-clamp success rate, defined in this study as achieving and maintaining a seal resistance above 200 MΩ, at 35°C could be as low as ~15% compared to ~80% at 25 and 15°C[31]. Thus, high temperature represents a limit to obtain

high success rates in patch-clamp experiments and will therefore preclude high-throughput evaluation of all hERG variants.

In this report, our aim was to optimize the functional characterization of hERG variants by circumventing these two opposite constraints (physiological temperature and seal resistance in the GΩ range). To do so, we adapted the AP-clamp protocol[24] in such a way that it could be used at room temperature to predict the reference repolarization power of hERG channels at 37°C.

Since hERG gating kinetics are slower at lower temperature, we attempted to mimic, at 10°C lower temperature, the hERG current profile observed at 37°C, by applying slower variation of voltage than the one used for AP-clamp at 37°C. Using AP-shaped voltage stimulations of various time scales applied at different temperatures, we determined that a unique correcting coefficient of 2 can be used to generate a current profile at room temperature that matches the one generated at physiological temperatures. We also illustrate that the repolarization power determined at room temperature is predictive of the repolarization power at physiological temperature for two pathogenic hERG variants with different biophysical dysfunctions.

Ultimately, we plan to use the repolarization power relative to the WT one to classify hERG variants. Large scale variant investigations using the pipeline we designed[24] will help to determine the limits for confidence interval for benign variants.

Material and methods

Cell culture

HEK293 cells were cultured in Dulbecco's modified Eagle's medium (Gibco, France) supplemented with 10% fetal calf serum (Eurobio, France), 4.5 g/L glucose, 2 mmol/L L-glutamine, 100 U/mL penicillin, 100 µg/mL and streptomycin (Corning, France) at 5% CO_2, maintained at 37°C in a humidified incubator. HEK293 cells stably expressing the human hERG channel (Bioprojet, France) were cultured in the same medium with 400 µg/mL G418 (Thermo Fisher Scientific, France), at 5% CO_2, maintained at 37°C in a humidified incubator. The cell lines were confirmed to be mycoplasma-free (MycoAlert, Lonza, France).

hERG plasmid transfection

In a first attempt of recording mutant hERG current using the automated patch-clamp system, HEK293 cells (passages 61-70) were transfected by electroporation using the MaxCyte STx system (MaxCyte Inc., MD, USA), using 30 µg plasmid per 100 µl Hyclone buffer[32]. For conventional patch-clamp experiments, the FuGENE 6 transfection reagent (Promega, WI, USA) was used to transfect wild-type (WT, protein sequence: NP_000229), p.R328C and p.D591H hERG plasmids. HEK293 cells (passages 24-30) were cotransfected, using 6 µL of

FuGENE 6, with 1.6 µg WT or mutant pcDNA5/FRT/TO Opti-hERG and 0.4 µg peGFP (Clontech, France) for fluorescence-based cell selection in microscopy[24]. Twenty-four hours after transfection, cells were trypsinized, diluted and plated on 35-mm dishes to obtain isolated cells for patch-clamp experiments.

High-throughput automated electrophysiology

Temperature effects on hERG channels were investigated on HEK293 cells stably expressing hERG (passages 28-33) using the automated patch-clamp system SyncroPatch 384PE (Nanion Technologies, Germany). Cells were detached with accutase (Innovative Cell Technologies, Inc., CA, USA) and floating single cells were diluted (~300,000 cells/mL) in medium containing (in mmol/L): NaCl 140, KCl 4.0, $CaCl_2$ 2.0, $MgCl_2$ 2.0, glucose 5.0 and HEPES 10 (pH 7.4, osmolarity 290 mOsm). Prior to recordings, dissociated cells were gently shaken at 200 rpm in a on a rotating platform at 10°C. NPC-384T 1x L-Type chips with typical single-hole and resistance of 2.40 ± 0.02 MΩ (n = 384) were used for recordings. Voltage stimulation and whole-cell recording were achieved using the PatchControl384 v1.5.3 software (Nanion Technologies) and the Biomek v1.0 interface (Beckman Coulter, France). The intracellular solution contained (in mmol/L): KCl 10, KF 110, NaCl 10, EGTA 10 and HEPES 10 (pH 7.2, osmolarity 280 mOsm), and, after seal formation, the extracellular solution contained (in mmol/L): NaCl 140, KCl 4.0, $CaCl_2$ 2.48, $MgCl_2$ 1.69, glucose 5.0 and HEPES 10 (pH 7.4, osmolarity 298 mOsm). After cell catch, seal, whole-cell formation and compensation, liquid application, and data acquisition were all performed sequentially and automatically. Whole-cell experiments were done at temperatures ranging from 22°C to 37°C. An AP stimulation protocol made of 6 voltage steps and ramps was designed to mimic the human ventricular sub-epicardial AP from the O'Hara and Rudy model[33] at 37°C (Figure S1).

Automated patch-clamp imposes the use of intracellular fluoride and high extracellular Ca^{2+} concentration (10 mmol/L) to obtain seals between the cell and the hole bored at the glass bottom of the recording well, close to 1 GΩ (Gigaseal). Extracellular Ca^{2+} concentration, after dilution, was kept relatively high (2.48 mmol/L) to maintain acceptable seal quality. To correct the screening effects of external Ca^{2+} on the surface charge of the membrane[34,35], and the uncompensated liquid junction potential[36], the stimulation potential was shifted by +32 mV. On the other hand, the holding potential was set to -80 mV, to allow full recovery from inactivation of hERG current (see Figure S2 for intermediary optimization steps, and Figure 1A, the dashed line representing the optimized AP). AP stimulation was repeated 4 times at a frequency of 1 Hz to ensure stability of the parameters.

In order to compensate for reduced hERG channel kinetics at temperatures lower than 37°C, and hence to allow larger current development during AP stimulation, the stimulation protocol

was adapted by applying a time factor. For example, when a factor of 2 was used, the AP duration was linearly doubled and the stimulation frequency slowed by 2 (see Figure 1B inset). Time factors varied from 0.5 to 5. Currents were sampled at 5 to 20 kHz. For each set of experiments, results were obtained with the same cell batch, on different plates (one per temperature), on the same day. Only recordings obtained with seal resistance (R_{seal}) > 500 MΩ, series resistance (R_s) < 10 MΩ, cell capacitance (C_m) > 10 pF, and with leak current between 0 and -200 pA at -80 mV before the 4th AP were considered for analysis, achieved with a custom semi-automated R routine. Currents are expressed as currents in pA. Before analyzing the current recordings, the leak current was automatically subtracted at each voltage value, after calculation from its measurement at resting potential, close to the equilibrium potential for K^+ ions. The repolarization power was calculated as the time integral of the absolute current during the 4th AP.

Conventional low-throughput electrophysiology

Temperature effects on WT and mutant hERG channels were also investigated on transfected HEK293 cells using conventional patch-clamp. One day after plating, transfected HEK293 cells were mounted on the stage of an inverted microscope and constantly perfused by a Tyrode solution first maintained at around 20.0°C, at a rate of 3 mL/min. HEPES-buffered Tyrode solution contained (in mmol/L): NaCl 145, KCl 4.0, $MgCl_2$ 1.0, $CaCl_2$ 1.0, HEPES 5.0, glucose 5.0, pH adjusted to 7.4 with NaOH. Patch pipettes (tip resistance: 2.0 to 2.5 MΩ) were pulled from soda lime glass capillaries (Kimble-Chase). The pipette was filled with an intracellular medium containing (in mmol/L): KCl 100, potassium gluconate 45, $MgCl_2$ 1.0, EGTA 5.0, HEPES 10, pH adjusted to 7.2 with KOH. Stimulation and data recording were performed with Clampex of pClamp 10 suite, an A/D converter (Digidata 1440A) and a Multiclamp 700B amplifier (all Axon Instruments, Molecular Devices, CA, USA)). Currents were acquired in the whole-cell configuration, filtered at 3 kHz and recorded at a sampling rate of 6.9 to 20 kHz. Before membrane capacitance and series resistance 70%-compensation, a series of twenty 30-ms steps to -80 mV was applied from a holding potential (HP) of alternatively -70 mV and -90 mV to subsequently off-line calculate C_m and R_s values from the recorded currents. A 3-step protocol was used to test the absence of current rundown/runup (HP =-80 mV, 1st pre-pulse: +40 mV during 1 s, 2nd pre-pulse: -100 mV during 15 ms, test-pulse: +40 mV during 500 ms, every 5 s). Then, an AP was used to clamp the voltage, that derived from the same O'Hara and Rudy model[33] as in automated patch-clamp. Unlike for automated patch-clamp, intracellular fluoride or high external Ca^{2+} concentration is not required to easily obtain Gigaseals. Therefore, the AP stimulation was corrected by the calculated liquid junctional potential only (see Figure 2A, the dashed line representing the AP stimulation)[24]. Three repeated APs were enough to stabilize the resulting I_{hERG} current time course. From around

20.0°C, Tyrode temperature was gradually raised until it reached 32.0°C in cell bath and AP-clamp recording was performed every 2.0°C. As in high-throughput experiments, in order to compensate for reduced hERG channel kinetics at lower temperatures, the stimulation protocol was adapted by applying a time factor, from 1 to 3: the same AP stimulation file (Axon text file: *.atf, compatible with Clampex) was used at various frequencies. We kept the typical quality control parameters of conventional patch-clamp to select the cells: R_{seal} > 1GΩ, R_s < 10 MΩ at the beginning and the end of the recording, and with less than 200 pA of leak current at -80 mV before the 3rd AP used to calculate the repolarization power. At the end of the experiment, the pipette potential offset was checked and recordings presenting offset drift > 5 mV were discarded. Currents are expressed as current densities in pA/pF. Before analyzing the current recordings, we subtracted the leak current, calculated at each voltage value from its measurement at resting potential. It has to be mentioned that only cells presenting detectable hERG currents were analyzed in order to be able to compare repolarization powers at various temperatures in the same cell. Data were analyzed and compiled using Clampfit of pClamp 10 suite, Microsoft Excel, and Prism (GraphPad Software, CA, USA). Statistical analyses were processed using Prism with Wilcoxon matched-pair signed rank test, or Mann-Whitney test, when appropriate. Significance level was set to 0.05.

Supplemental results

Temperature dramatically affects the success rate in high-throughput automated patch-clamp system

To illustrate the impact of temperature on the success rate of hERG recordings, we first investigated the seal quality quantified with its resistance (R_{seal}) as a function of temperature using stable cell lines expressing hERG channels. For that purpose, we used the automated patch-clamp system that provides robust information on the temperature effect by dint of great numbers of cells that can be recorded at once. As illustrated in Figure S3A, the percentage of HEK293 cells with high-quality R_{seal} ≥ 1 GΩ decreased significantly when the recording temperature is increased. The success rate dropped from 38 to 13% when measured at 27 and 37°C, respectively. In this case, one could consider that the success rate at 37°C is acceptable although very low. However, if one aims to characterize hundreds of hERG variants, the most realistic approach is to use transient expression which may add another fragilizing factor to seal quality. To test this, we electroporated HEK293 cells with a plasmid over-expressing hERG and estimated again the effect of temperature on seal quality. The percentage of cells reaching a R_{seal} ≥ 1 GΩ at 27°C reached 18% but only 21 cells (5.4% of the plate wells) exhibited hERG currents above 50 pA at +50 mV (Figure S3C). In contrast, at 37°C, the percentage of cells with R_{seal} ≥ 1 GΩ dropped to 5% and none of them displayed any hERG current. Including cells with R_{seal} < 1 GΩ but ≥ 500 MΩ improved marginally only the

rate of cells presenting measurable K^+ currents (3 cells at 37°C - Figure S3B). On the other hand, for HEK293 cells stably expressing hERG channels, the success rate of 38% at 27°C increased to 77% when the seal resistance limit dropped from 1 GΩ to 500 MΩ. Of note, this seal quality remains largely above previously accepted ones by others[29,31] during automated patch-clamp experiments using stable cell lines (≥ 100 and 200 MΩ, respectively).

However, even the medium-quality experimental conditions with seal resistance values above 500 MΩ are not sufficient to reasonably increase data production yield at physiological temperature if one desires to tackle the biophysical properties of hundreds of hERG variants. Thus, an alternative protocol is needed to predict at room temperature the *in vivo* impact of variants. As validation of this new protocol, we compared currents obtained at room and physiological temperatures using automated patch-clamp system for HEK293 cells stably expressing hERG channels and conventional patch-clamp technique for cells transiently over-expressing hERG variants, choosing, in this latter case, gold-standard quality criteria to accurately evaluate the temperature effects.

Limitations

The validation of the correcting value used for a 10°C decrease has been conducted during AP clamp in specific time- and voltage-frames, the readout being the repolarization power preservation. The time factor that we are using is not predictive of the Q_{10} of each biophysical parameter but may be regarded as a global correcting value. Further tests would be necessary to challenge the correcting value stability with prolonged AP or in depolarized conditions.

As for the ILT variant of Shaker, another voltage-gated channel, some missense mutations may alter the channel sensitivity to temperature[37]. This would prevent to predict the repolarization power, just by applying the time correcting value of 2. However, such an alteration, due to uncoupling of the voltage sensor from the activation gate is also associated with dramatic changes in the activation voltage-dependence of ILT Shaker[38]. From this observation, we can hypothesize that a hERG variant exhibiting a similar prominent uncoupling between the voltage sensor and the activation gate, would barely activate during the action potential whatever the experimental temperature.

Milnes et al. proposed AP clamp on hERG at physiological temperature to be part of safety testing of novel drug candidates[39] allowing to integrate the drug dissociation/reassociation kinetics in the same voltage protocol. It may be then of interest to use the repolarization index instead of the current amplitude as readout for a more 'physiological IC_{50} value determination. However, if the correcting value of 2 may compensate for the temperature effect on hERG channel behavior, it may not be adapted to binding and unbinding kinetics changes.

Supplemental figures

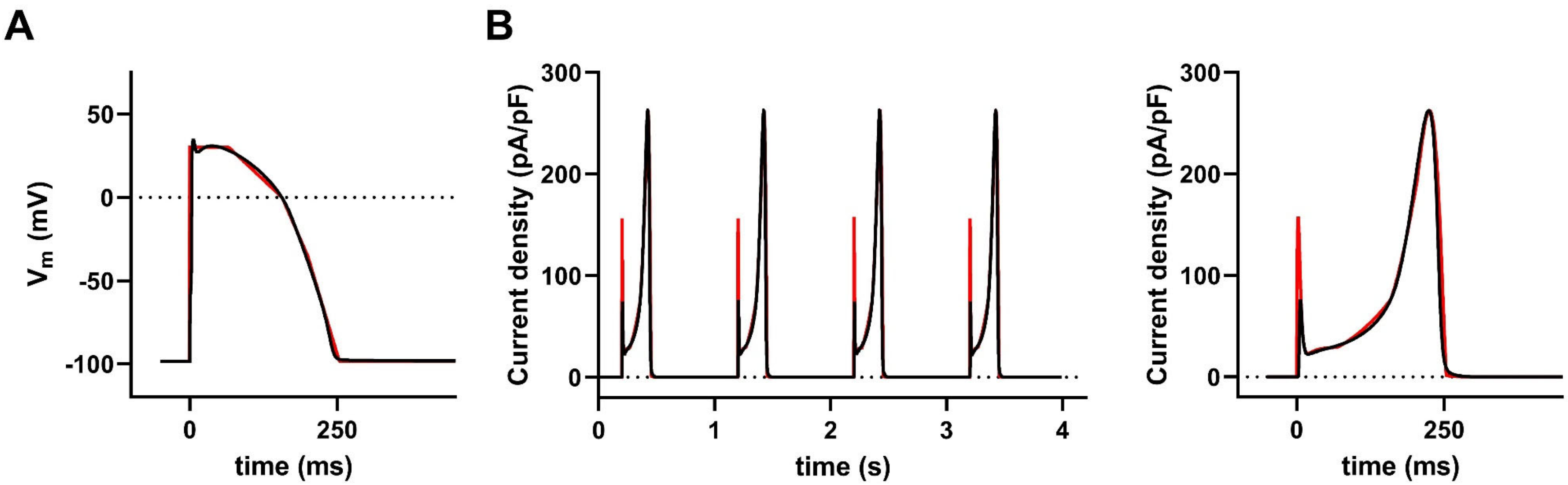

Figure S1: The I_{Kr} current (B, red), generated *in silico*[40] using the simplified AP (A, red) as stimulation, adequately overlaps with the one (B, black) using the human ventricle AP generated by O'Hara and Rudy model (A, black)[33].

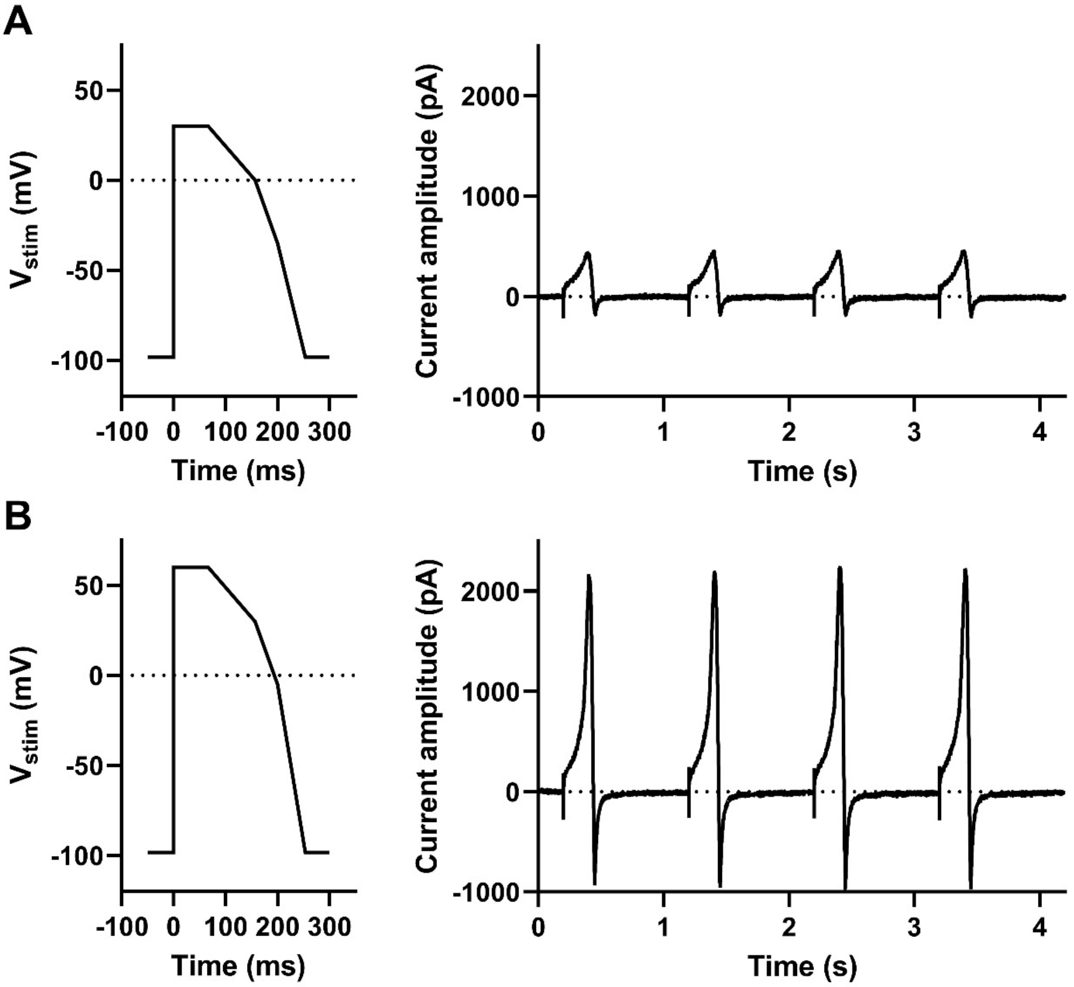

Figure S2: Optimization of the AP stimulation protocol for automated patch clamp. (A) Original AP (made of 6 voltage steps and ramps) mimicking the human sub-epicardial ventricular AP from the O'Hara and Rudy model[33]. (B) After correction of the screening effects of external Ca^{2+} on the surface charge of the membrane and compensation of the liquid junction potential, the higher overshoot allowed development of a much larger K^+ current (all recordings at 37°C, same cell as A). In this example, the holding potential (HP) was still set at -100 mV and a transient inward current due to hERG deactivation was recorded at the end of the AP repolarization. Therefore, HP of the final AP stimulation protocol was set to -80 mV, limiting the inward K^+ current but still allowing full recovery of hERG current.

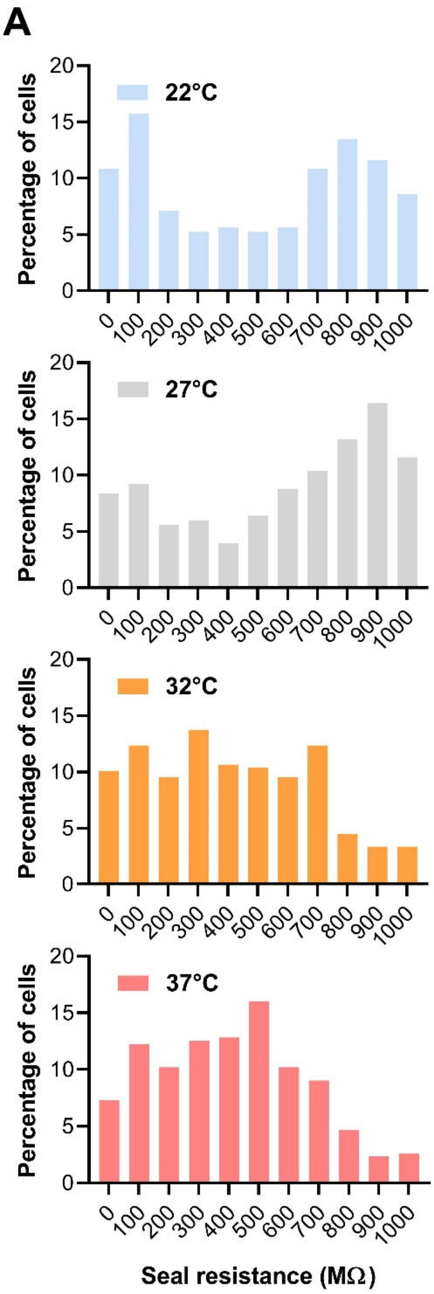

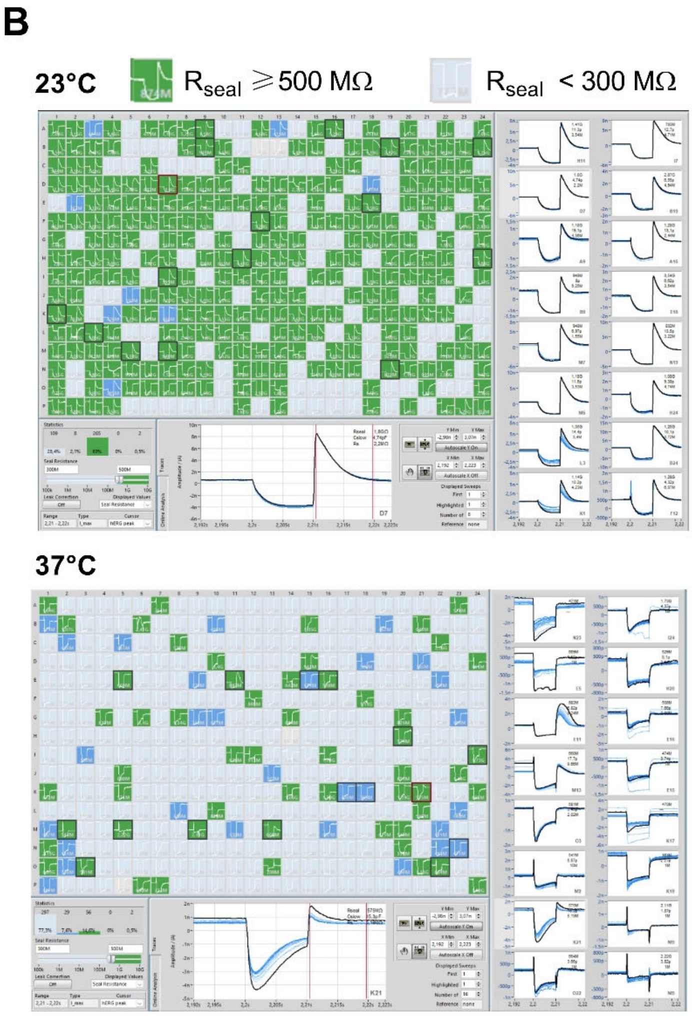

C

Automated patch clamp (384 cells/plate)		27°C	37°C	27°C	37°C
		$R_{seal} \geq 1$ GΩ		$R_{seal} \geq 500$ MΩ	
Stable HEK-293 cell line	%	38%	13%	77%	42%
	(n)	144	48	294	160
Electroporated HEK-293 cells	%	5%	0%	10%	1%
	(n)	21	0	40	3

Figure S3: Evolution of the seal resistance (R_{seal}) with increasing temperatures. (A) R_{seal} value distribution for HEK293 cells stably expressing WT hERG channels (one 384-well plate per temperature) using automated patch-clamp system. (B) Graphical user interface illustrating single-cell recordings from transiently transfected HEK293 cells at 23 and 37°C, 30 h post-transfection (electroporated cells, one 384-well plate per temperature). Each recording well is visualized and color-coded based on user-defined quality criteria. A single-cell recording is considered as successful when $R_{seal} \geq 500$ MΩ (green). (C) Summary of percentage of cell with measurable hERG current in various conditions. The dramatic drop of recordings with $R_{seal} \geq 500$ MΩ at 37°C precludes the hERG current measurements at physiological temperature on transiently transfected cells using automated patch-clamp system.

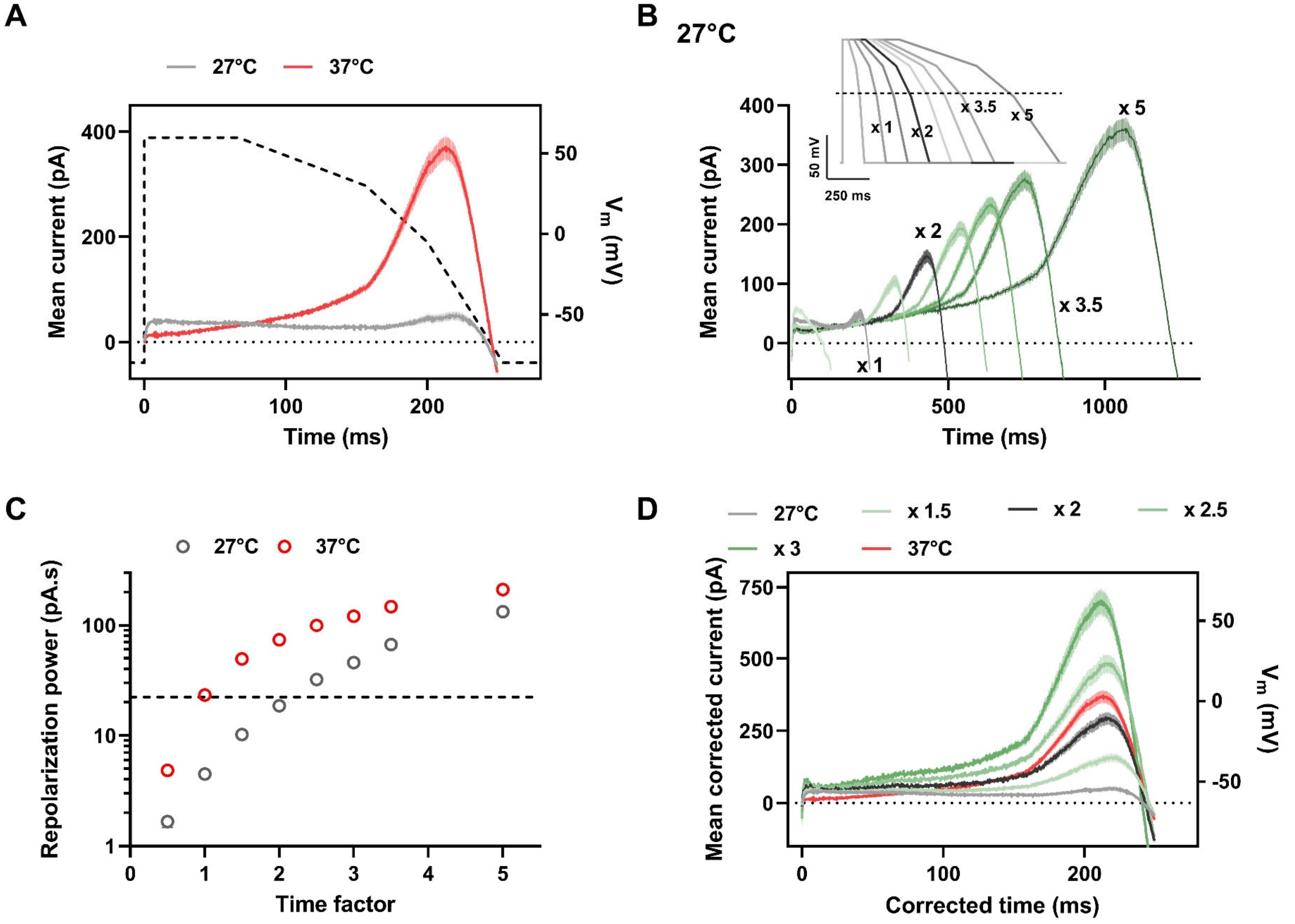
A
27°C
37°C
Mean current (pA)
Vm (mV)
Time (ms)
B
27°C
x 1
x 2
x 3.5
x 5
50 mV
250 ms
Mean current (pA)
Time (ms)
C
27°C
37°C
Repolarization power (pA.s)
Time factor
D
27°C
x 1.5
x 2
x 2.5
x 3
37°C
Mean corrected current (pA)
Vm (mV)
Corrected time (ms)

Figure S4: Repolarization power of WT hERG as a function of temperature – Replicate. A simplified action potential was applied (AP-clamp) on HEK293 cells stably expressing WT hERG by automated patch clamp system in 384-well plates. (A) Mean (± SEM) current recordings during AP-clamp at 27 and 37°C (in pA/pF, n = 123 and 150 cells, respectively). Dashed line: time course of the simplified AP (voltage scale: right Y axis). (B) Mean (± SEM) current recordings during AP-clamp at 27°C for AP of various durations (see inset for APs; n = 59-230 for Time x 0.5 to x 5). (C) Mean (± SEM) repolarization power at 27 and 37°C *vs.* time factor (n = 59-230 and 107-158, respectively). Horizontal dashed line: repolarization power at 37°C: 23.2 pA/pF.s. (D) As in (B), at 27°C, after time and current corrections using various factors from 1.5 to 3 on the respective recordings. For the recordings obtained during the AP of x 2 duration, the time was divided by 2 and the current multiplied by 2. Note the overlap of the current corrected by the factor of 2 at 27°C (black) with the reference current obtained during the standard AP at 37°C (red).

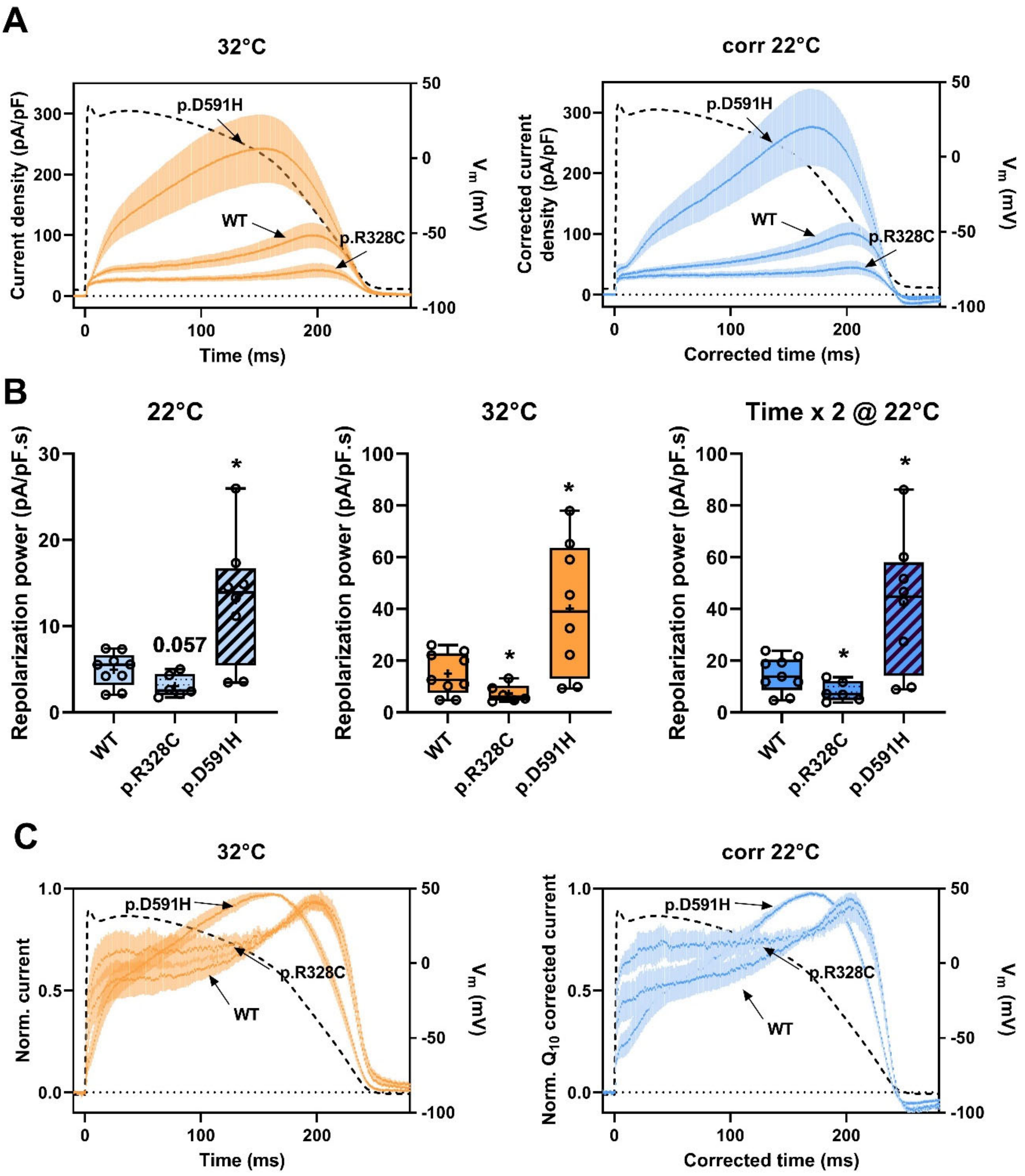

Figure S5: Repolarization power of WT, LQTS (p.R328C) and SQTS (p.D591H) hERG as a function of temperature – Comparison. AP clamp was applied on HEK293 cells transiently expressing WT or hERG variants (same cells as Figures 2 and 3). (A) Mean (± SEM) current recordings during AP-clamp at 32°C (orange; n = 9, 6 and 8 for WT, p.R328C and p.D591H, respectively) and for AP of 2x duration at 22°C after time and current density corrections using the coefficient of 2 on the respective recordings (blue). (B) Tukey plots of the repolarization powers recorded at 32 and 22°C during standard or AP of doubled duration. *vs*. WT: *: $P < 0.05$ or P value when non-significant. (C) As in (A) after normalization to the maximum current density value.

Supplemental references

1. Priori SG, Blomström-Lundqvist C, Mazzanti A, et al. 2015 ESC Guidelines for the management of patients with ventricular arrhythmias and the prevention of sudden cardiac death: The Task Force for the Management of Patients with Ventricular Arrhythmias and the Prevention of Sudden Cardiac Death of the European Society of Cardiology (ESC)Endorsed by: Association for European Paediatric and Congenital Cardiology (AEPC). *Eur Heart J*. 2015;36(41):2793-2867. doi:10.1093/eurheartj/ehv316

2. Krahn AD, Laksman Z, Sy RW, et al. Congenital Long QT Syndrome. *JACC Clin Electrophysiol*. 2022;8(5):687-706. doi:10.1016/j.jacep.2022.02.017

3. Roden DM, Viswanathan PC. Genetics of acquired long QT syndrome. *J Clin Invest*. 2005;115(8):2025-2032. doi:10.1172/JCI25539

4. For Research Scientists :: Crediblemeds. Accessed January 20, 2023. https://www.crediblemeds.org/research-scientists

5. Schwartz PJ, Priori SG, Spazzolini C, et al. Genotype-phenotype correlation in the long-QT syndrome: gene-specific triggers for life-threatening arrhythmias. *Circulation*. 2001;103(1):89-95. doi:10.1161/01.cir.103.1.89

6. Kolder ICRM, Tanck MWT, Postema PG, et al. Analysis for Genetic Modifiers of Disease Severity in Patients With Long-QT Syndrome Type 2. *Circ Cardiovasc Genet*. 2015;8(3):447-456. doi:10.1161/CIRCGENETICS.114.000785

7. Kutyifa V, Daimee UA, McNitt S, et al. Clinical aspects of the three major genetic forms of long QT syndrome (LQT1, LQT2, LQT3). *Ann Noninvasive Electrocardiol*. 2018;23(3):e12537. doi:10.1111/anec.12537

8. Itoh H, Crotti L, Aiba T, et al. The genetics underlying acquired long QT syndrome: impact for genetic screening. *Eur Heart J*. 2016;37(18):1456-1464. doi:10.1093/eurheartj/ehv695

9. Charpentier F, Mérot J, Loussouarn G, Baró I. Delayed rectifier K+ currents and cardiac repolarization. *J Mol Cell Cardiol*. 2010;48(1):37-44. doi:10.1016/j.yjmcc.2009.08.005

10. Roden DM. Taking the "Idio" out of "Idiosyncratic": Predicting Torsades de Pointes. *Pacing Clin Electrophysiol*. 1998;21(5):1029-1034. doi:10.1111/j.1540-8159.1998.tb00148.x

11. kcnh2[gene] "long QT" - ClinVar - NCBI. Accessed January 20, 2023. https://www.ncbi.nlm.nih.gov/clinvar/?term=kcnh2%5Bgene%5D+%22long+QT%22

12. Gaita F, Giustetto C, Bianchi F, et al. Short QT Syndrome: A Familial Cause of Sudden Death. *Circulation*. 2003;108(8):965-970. doi:10.1161/01.CIR.0000085071.28695.C4

13. Brugada R, Hong K, Dumaine R, et al. Sudden Death Associated With Short-QT Syndrome Linked to Mutations in HERG. *Circulation*. 2004;109(1):30-35. doi:10.1161/01.CIR.0000109482.92774.3A

14. Anderson CL, Kuzmicki CE, Childs RR, Hintz CJ, Delisle BP, January CT. Large-scale mutational analysis of Kv11.1 reveals molecular insights into type 2 long QT syndrome. *Nat Commun*. 2014;5(1):5535. doi:10.1038/ncomms6535

15. Ng CA, Perry MD, Liang W, et al. High-throughput phenotyping of heteromeric human ether-à-go-go-related gene potassium channel variants can discriminate pathogenic from rare benign variants. *Heart Rhythm*. 2020;17(3):492-500. doi:10.1016/j.hrthm.2019.09.020

16. Alameh M, Oliveira-Mendes BR, Kyndt F, et al. A need for exhaustive and standardized characterization of ion channels activity. The case of KV11.1. *Front Physiol*. 2023;14. Accessed April 24, 2023. https://www.frontiersin.org/articles/10.3389/fphys.2023.1132533

17. Richards S, Aziz N, Bale S, et al. Standards and guidelines for the interpretation of sequence variants: a joint consensus recommendation of the American College of Medical Genetics and Genomics and the Association for Molecular Pathology. *Genet Med Off J Am Coll Med Genet*. 2015;17(5):405-424. doi:10.1038/gim.2015.30

18. Base de données BAMACOEUR. Cardiogen. Accessed September 14, 2022. https://www.filiere-cardiogen.fr/bamacoeur/

19. Bellocq C, Wilders R, Schott JJ, et al. A common antitussive drug, clobutinol, precipitates the long QT syndrome 2. *Mol Pharmacol*. 2004;66(5):1093-1102. doi:10.1124/mol.104.001065

20. Jouni M, Si-Tayeb K, Es-Salah-Lamoureux Z, et al. Toward Personalized Medicine: Using Cardiomyocytes Differentiated From Urine-Derived Pluripotent Stem Cells to Recapitulate Electrophysiological Characteristics of Type 2 Long QT Syndrome. *J Am Heart Assoc*. 2015;4(9):e002159. doi:10.1161/JAHA.115.002159

21. Brandão KO, van den Brink L, Miller DC, et al. Isogenic Sets of hiPSC-CMs Harboring Distinct KCNH2 Mutations Differ Functionally and in Susceptibility to Drug-Induced Arrhythmias. *Stem Cell Rep*. 2020;15(5):1127-1139. doi:10.1016/j.stemcr.2020.10.005

22. Chevalier P, Rodriguez C, Bontemps L, et al. Non-invasive testing of acquired long QT syndrome: evidence for multiple arrhythmogenic substrates. *Cardiovasc Res*. 2001;50(2):386-398. doi:10.1016/s0008-6363(01)00263-2

23. Anderson CL, Delisle BP, Anson BD, et al. Most LQT2 Mutations Reduce Kv11.1 (hERG) Current by a Class 2 (Trafficking-Deficient) Mechanism. *Circulation*. 2006;113(3):365-373. doi:10.1161/CIRCULATIONAHA.105.570200

24. Oliveira-Mendes B, Feliciangeli S, Ménard M, et al. A standardised hERG phenotyping pipeline to evaluate KCNH2 genetic variant pathogenicity. *Clin Transl Med*. 2021;11(11):e609. doi:10.1002/ctm2.609

25. Zhou Z, Gong Q, Ye B, et al. Properties of HERG Channels Stably Expressed in HEK 293 Cells Studied at Physiological Temperature. *Biophys J*. 1998;74(1):230-241. doi:10.1016/S0006-3495(98)77782-3

26. Vandenberg JI, Varghese A, Lu Y, Bursill JA, Mahaut-Smith MP, Huang CLH. Temperature dependence of human *ether-à-go-go-* related gene K^+ currents. *Am J Physiol-Cell Physiol*. 2006;291(1):C165-C175. doi:10.1152/ajpcell.00596.2005

27. Li Z, Dutta S, Sheng J, Tran PN, Wu W, Colatsky T. A temperature-dependent in silico model of the human ether-à-go-go-related (hERG) gene channel. *J Pharmacol Toxicol Methods*. 2016;81:233-239. doi:10.1016/j.vascn.2016.05.005

28. Mauerhöfer M, Bauer CK. Effects of Temperature on Heteromeric Kv11.1a/1b and Kv11.3 Channels. *Biophys J*. 2016;111(3):504-523. doi:10.1016/j.bpj.2016.07.002

29. Lei CL, Clerx M, Gavaghan DJ, Polonchuk L, Mirams GR, Wang K. Rapid Characterization of hERG Channel Kinetics I: Using an Automated High-Throughput System. *Biophys J*. 2019;117(12):2438-2454. doi:10.1016/j.bpj.2019.07.029

30. Lei CL, Clerx M, Beattie KA, et al. Rapid Characterization of hERG Channel Kinetics II: Temperature Dependence. *Biophys J*. 2019;117(12):2455-2470. doi:10.1016/j.bpj.2019.07.030

31. Ranjan R, Logette E, Marani M, et al. A Kinetic Map of the Homomeric Voltage-Gated Potassium Channel (Kv) Family. *Front Cell Neurosci*. 2019;13:358. doi:10.3389/fncel.2019.00358

32. Vanoye CG, Desai RR, Fabre KL, et al. High-Throughput Functional Evaluation of *KCNQ1* Decrypts Variants of Unknown Significance. *Circ Genomic Precis Med*. 2018;11(11):e002345. doi:10.1161/CIRCGEN.118.002345

33. O'Hara T, Virág L, Varró A, Rudy Y. Simulation of the Undiseased Human Cardiac Ventricular Action Potential: Model Formulation and Experimental Validation. McCulloch AD, ed. *PLoS Comput Biol*. 2011;7(5):e1002061. doi:10.1371/journal.pcbi.1002061

34. Hille B. Ionic channels in excitable membranes. Current problems and biophysical approaches. *Biophys J*. 1978;22(2):283-294. doi:10.1016/S0006-3495(78)85489-7

35. Ho WK, Kim I, Lee CO, Earm YE. Voltage-dependent blockade of HERG channels expressed in *Xenopus* oocytes by external Ca $^{2+}$ and Mg $^{2+}$. *J Physiol*. 1998;507(3):631-638. doi:10.1111/j.1469-7793.1998.631bs.x

36. Barry PH, Lynch JW. Liquid junction potentials and small cell effects in patch-clamp analysis. *J Membr Biol*. 1991;121(2):101-117. doi:10.1007/BF01870526

37. Yang F, Zheng J. High temperature sensitivity is intrinsic to voltage-gated potassium channels. Aldrich R, ed. *eLife*. 2014;3:e03255. doi:10.7554/eLife.03255

38. Smith-Maxwell CJ, Ledwell JL, Aldrich RW. Uncharged S4 Residues and Cooperativity in Voltage-dependent Potassium Channel Activation. *J Gen Physiol*. 1998;111(3):421-439. doi:10.1085/jgp.111.3.421

39. Milnes JT, Witchel HJ, Leaney JL, Leishman DJ, Hancox JC. Investigating dynamic protocol-dependence of hERG potassium channel inhibition at 37°C: Cisapride versus dofetilide. *J Pharmacol Toxicol Methods*. 2010;61(2):178-191. doi:10.1016/j.vascn.2010.02.007

40. ten Tusscher KHWJ, Noble D, Noble PJ, Panfilov AV. A model for human ventricular tissue. *Am J Physiol-Heart Circ Physiol*. 2004;286(4):H1573-H1589. doi:10.1152/ajpheart.00794.2003
